# Experimental sleep loss, racial bias, and the decision criterion to shoot in the Police Officer’s Dilemma task

**DOI:** 10.1038/s41598-020-77522-z

**Published:** 2020-11-25

**Authors:** Michael K. Scullin, Michelle R. Hebl, Abby Corrington, Stacy Nguyen

**Affiliations:** 1 Department of Psychology and Neuroscience, Baylor University, One Bear Place 97334, Waco, TX 76798 USA; 2 Department of Psychological Sciences, Rice University, Houston, TX USA; 3 Department of Management, Providence College School of Business, Providence, RI USA; 4 School of Medicine, University of Texas Health Sciences Center, San Antonio, TX USA

**Keywords:** Cognitive control, Human behaviour

## Abstract

Violent behavior, police brutality, and racial discrimination are currently at the forefront of society’s attention, and they should be. We investigated whether mild sleep loss—as typical for many adults throughout the work week—could aggravate the socio-emotional-cognitive processes contributing to violence and discrimination. In a sample of 40 healthy young adults, we either experimentally restricted participants’ sleep for four nights (6.2 h/night) or let participants obtain normal sleep (7.7 h/night)—and then had them complete the Police Officer’s Dilemma Task. In this computerized task, the participant must rapidly decide to shoot or not shoot at White and Black men who either are or are not holding a gun. Results showed significant racial biases, including more and quicker shooting of Black targets compared to White targets. Furthermore, signal detection analyses demonstrated that mild sleep restriction changed participants’ decision criterion, increasing the tendency to shoot, even when controlling for psychomotor vigilance, fluid intelligence, and self-reported desirability to behave in a socially acceptable manner. The increased tendency to shoot was also observed in participants who reported believing that they had adapted to the sleep loss. Future experimental research using trained police officers will help establish the generalizability of these laboratory effects. Importantly, sleep loss is modifiable via organization-level changes (e.g., shift scheduling, light entrainment) and individual-level interventions (e.g., sleep hygiene education, incentives for behavioral change), suggesting that if sleep loss is corrected, it could save lives—including Black lives.

## Introduction

On the night of September 6, 2018, a White police officer returned to what she believed was her apartment, opening the door to find Mr. Botham Jean, a Black man. The officer, Amber Guyger, quickly pulled her gun and shot him in the chest, killing him. Botham Jean was unarmed and simply eating ice cream in his own—not the officer’s—apartment^1^. At the trial, it was revealed that the police officer was “fatigued” from having worked 40 h in the previous four days, including just finishing a shift that lasted at least 13 h^2^. While there are many racial bias-related components of the Guyger/Jean case and other police shootings that warrant empirical investigation, the observation that Guyger was “fatigued” at the time of the shooting should be of particular interest to sleep researchers.

Chronic sleep loss has been called an “epidemic”^3^, with some estimating that sleep in United States citizens has dropped from nearly 8 h/night in the 1940s to 7 or fewer hours/night more recently^3,4^. Sleep loss is common among people of many different professions, but because police officers carry firearms and often must handle potentially hostile situations, it is crucial to recognize that their shifts can be particularly long, variable, and circadian-misaligned^5^. In a large, representative sample, 40% of police officers had clinical-level sleep disorders and more than ¼ of officers reported falling asleep while driving at least once per month^6^. Such widespread sleep loss—whether in police officers or any other citizen—is likely to be consequential. After all, acute and chronic sleep loss are known to have numerous neurobiological consequences for nearly all people (i.e., only a small percentage of the population can maintain short sleep without consequences^7^).

In the current work, we investigated sleep loss as a potent mechanism for altering socio-emotional-cognitive processes, which could then correspond to increased expressions of biases and discrimination^8^. Discrimination is, of course, driven by prejudice and racism, but there are at least three additional mechanisms by which sleep deprivation could contribute to when and how discrimination manifests. First, sleep deprivation can alter frontoparietal attentional network functioning^9,10^. For decades, researchers have known that sleep restriction slows responding and increases lapses of attention^11,12^. Relatedly, if one’s attentional resources are depleted, then they may lack the ability to inhibit prejudices they hold that are typically suppressed^13^. Second, sleep deprivation can increase emotional reactivity. In one experiment, participants either slept normally or were sleep deprived and then shown negative images (e.g., snake attacking, person holding a gun). Sleep deprivation increased amygdala reactivity to the negative images due to decreased connectivity between the medial prefrontal cortex and the amygdala^14^. Third, sleep deprivation can alter threat perceptions. In a recent study, participants who either were sleep deprived or had normal sleep were shown a series of faces and asked to classify them as threatening or non-threatening^15^. Following sleep deprivation, more faces were rated as threatening, a pattern that corresponded to activity in the salience detection network^8^. Whether sleep deprivation’s effects on attention, emotional reactivity, or threat perception translate to greater discrimination following sleep loss has received minimal empirical study and remains poorly understood^16–21^.

## Results

To mirror the chronic mild sleep loss experienced by many individuals during the work week, we randomly assigned 40 healthy adult participants (ages 18–27, 66.7% female, 52.4% White, 31.0% Asian, 14.3% Hispanic, 2.4% Mixed, 0% Black) to maintain a bedtime of either 10:30 pm or 1:30 am from Monday to Friday^22^. Waketime was set to 7:30 am each day. By using sealed envelopes and blocked randomization, experimenters and analysts remained blinded to participants’ randomly assigned normal versus restricted sleep conditions. The normal sleep condition averaged 1.5 h more sleep per night than the restricted sleep condition, *t*(38) = 5.87, *p* < 0.001, *d* = 1.85 (Table 1). Furthermore, by Friday, the sleep restricted participants experienced greater daytime sleepiness [*t*(38) = 4.02, *p* < 0.001, *d* = 1.27], greater moodiness [*t*(38) = 2.40, *p* = 0.021, *d* = 0.76], and poorer sustained attention (assessed using the psychomotor vigilance task; *t*(38) = 2.16, *p* = 0.037, *d* = 0.68; Table 1). We did not measure explicit prejudices to avoid demand characteristics.

**Table 1:** Sample characteristics and task performance.

	Restricted sleep(n = 20)	Normal sleep(n = 20)	Statistic
**Monday measures (session 1)**			
Age (years)	21.20 ± 2.19	20.25 ± 1.48	*t*(38) = 1.61, *p* = .12
Gender (% female)	60%	70%	X^2^(1) = 0.44, *p* = .51
Race/ethnicity (%White)	55%	45%	X^2^(1) = 0.40, *p* = .53
Social Desirability Scale	17.50 ± 4.90	15.85 ± 4.60	*t*(38) = 1.097, *p* = .28
Pittsburgh Sleep Quality Index	5.25 ± 2.51	5.25 ± 2.27	*t*(38) < .01, *p* > .99
Karolinska Sleepiness Scale	4.35 ± 1.79	5.21 ± 1.55	*t*(38) = 1.60, *p* = .12
Profile of mood states − total mood disturbance	92.10 ± 17.16	90.25 ± 11.76	*t*(38) = 0.40, *p* = .69
**Friday measures (session 2)**			
Total sleep time (actigraphy minutes)	372.56 ± 52.95	461.73 ± 42.64	*t*(38) = 5.87, *p* < .001
Perceived they adapted to sleep loss (%)	40%	–-	
Karolinska Sleepiness Scale	5.30 ± 1.83	3.15 ± 1.53	*t*(38) = 4.02, *p* < .001
Profile of mood states − total mood disturbance	111.35 ± 24.74	94.90 ± 18.06	*t*(38) = 2.40, *p* = .021
Psychomotor vigilance task–response times	325.13 ± 50.02	294.07 ± 40.27	*t*(38) = 2.16, *p* = .037
Raven’s progressive matrices (of 18)	10.80 ± 3.44	11.10 ± 2.55	*t*(38) = 0.76, *p* = .76

Data are plotted as mean ± standard deviation, with measures separated by Monday (Session 1) and Friday (Session 2) data collection. The conditions did not differ pre-experimentally (Session 1), but showed strong differences in sleep duration, daytime sleepiness, mood disturbances, and vigilance by the end of the week.

The Friday test session^22^ concluded with the Police Officer’s Dilemma Task^23,24^. This task was developed to simulate the real-world challenge of police officers needing to make quick decisions in response to perceptually ambiguous situations (i.e., threatening or non-threatening). Participants were shown scenes that were either empty (no person) or contained a target (person). The target person was either unarmed or armed with a gun and either White or Black, and participants had to quickly decide whether to shoot or not shoot the target. We analyzed accuracy, reaction times, timeouts (failures to respond within the time limit), and signal-detection-theory metrics by conducting a series of 2 × 2 analyses of variance (ANOVAs) that included target race (White, Black) and sleep condition (normal, restricted).

**Figure 1:**
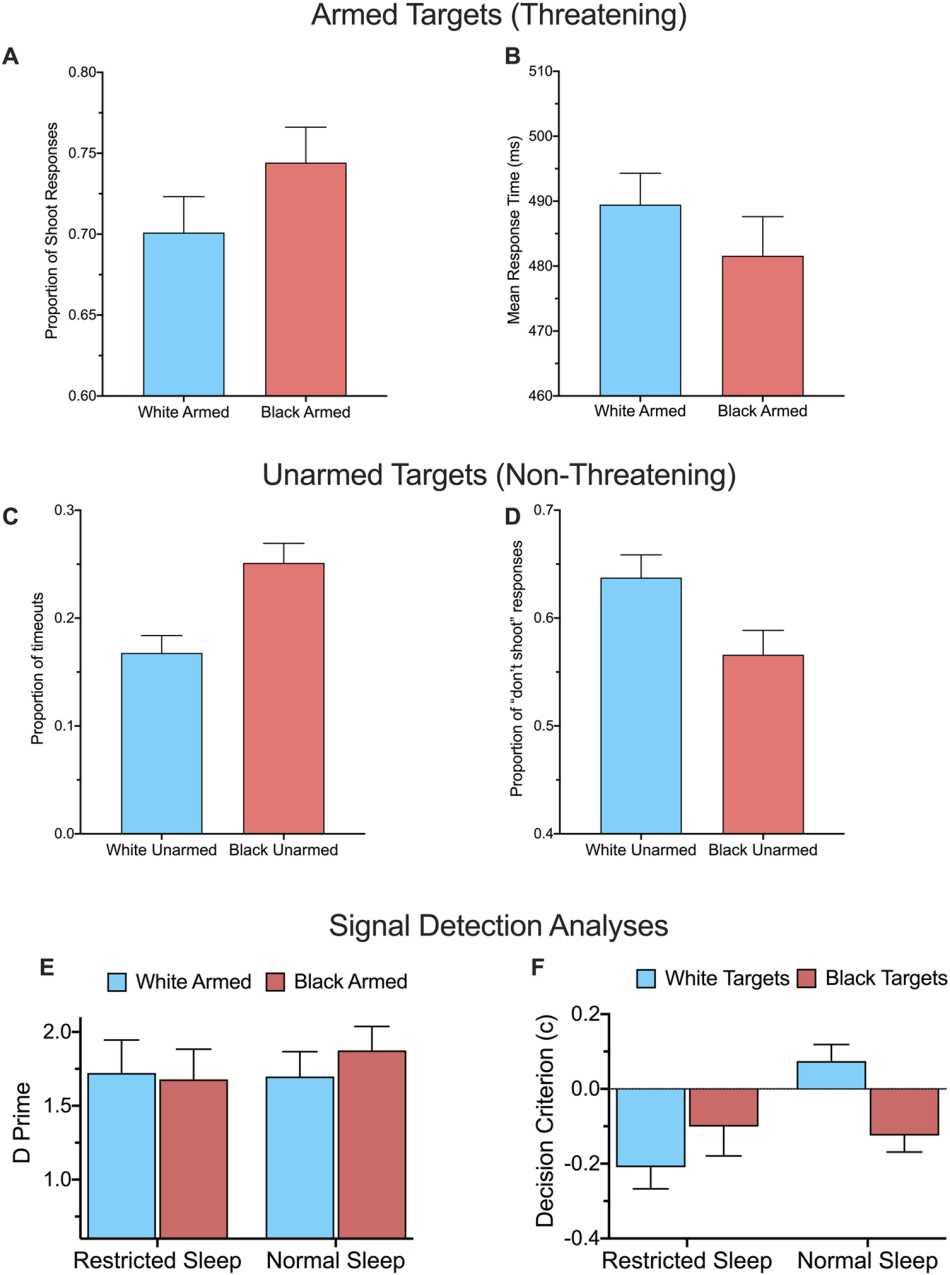
Performance on the Police Officer’s Dilemma Task. There was evidence for racial bias in response latencies and shoot responses on threatening target trials (**A**, **B**) as well as indecision and absence of non-shoot responses on non-threating target trials (**C**, **D**). Sleep restriction was unrelated to *d*′ sensitivity (E), but altered the decision criterion for shooting toward leniency (**F**). Data shown as mean ± SEM.

The results revealed significant evidence for racial biases. On armed trials, participants were more likely to “shoot” Black targets than White targets (Fig. 1A), *F*(1, 38) = 5.37, *p* = 0.026, η_p_^2^ = 0.12, and with quicker response times (Fig. 1B), *F*(1, 38) = 4.13, *p* = 0.049, η_p_^2^ = 0.10. On unarmed trials, there were no effects for mean response times (*p*s > 0.10), but there were far more failures to respond within time limits (timeouts) for Black targets than White targets (Fig. 1C), *F*(1, 38) = 19.63, *p* < 0.001, η_p_^2^ = 0.34. This result suggests that participants experienced greater difficulty in quickly identifying the Black targets as non-threatening. Similarly, there were significantly fewer “don’t shoot” responses to unarmed Black targets than unarmed White targets (Fig. 1D), *F*(1, 38) = 11.45, *p* = 0.002, η_p_^2^ = 0.23. False alarms were rare and showed nonsignificant effects. We conducted follow-up analyses to determine whether any of the significant effects interacted with gender. All gender main effects and interactions were nonsignificant (*ps* > 0.05), indicating that men and women demonstrated similar levels of racial biases in the Police Officer’s Dilemma Task. The averaged accuracy and averaged response time metrics were not significantly affected or moderated by sleep condition (*p*s > 0.05).

An effect of sleep condition emerged when conducting analyses based on signal detection theory. We distinguished sensitivity to armed targets (*d*′) and the decision criterion (*c*) for shooting a target. Similar to previous reports^24^, there were no significant effects on *d*′ (Fig. 1E; *p*s > 0.10). Importantly, for *c*, there was a significant main effect of sleep condition such that sleep restriction (*M* = − 0.155) caused greater leniency in shooting targets relative to normal sleep (*M* = − 0.025), *F*(1, 38) = 4.63, *p* = 0.038, η_p_^2^ = 0.11. Criterion scores were significantly lower than zero after sleep restriction, *t*(19) = 3.20, *p* = 0.005, *d* = 1.47, but did not differ significantly from zero after normal sleep, *t*(19) = 0.49, *p* = 0.49, *d* = 0.22. Figure 1F further illustrates that sleep condition interacted with target race, *F*(1, 38) = 7.75, *p* = 0.008, η_p_^2^ = 0.17. Being well-rested led to a more conservative criterion for White targets, *t*(38) = 3.89, *p* < 0.001, *d* = 1.23, but this benefit of rested sleep did not extend to Black targets, *t*(38) = 0.27, *p* = 0.79, *d* = 0.09. In other words, all groups showed a lenient bias except for well-rested participants’ responses to White targets. The interaction remained statistically significant even when controlling for participants’ psychomotor vigilance, fluid intelligence, self-reported desirability to behave in a socially acceptable manner, and self-reported belief that they had adapted to the sleep loss, *F*(1, 34) = 6.07, *p* = 0.019, η_p_^2^ = 0.15.

The effects of sleep restriction and target race on decision criterion were not moderated by gender, *F*(1, 36) = 0.74, *p* = 0.40, η_p_^2^ = 0.02 (and there were no interactions; all *Fs* < 1). Women and men showed identically lenient *c* scores after sleep restriction [*M*s = − 0.158 and − 0.151, respectively, *F*(1, 18) = 0.004, *p* = 0.95, η_p_^2^ < 0.01] and similar *c* scores after normal sleep (*Ms* = − 0.056 and 0.048, respectively, *F*(1, 18) = 1.84, *p* = 0.19, η_p_^2^ = 0.09). Interestingly, when limiting the sample to only White participants, the sleep restriction effects on decision criterion were exacerbated. For example, the effect size increased from η_p_^2^ = 0.11 in the overall sample to η_p_^2^ = 0.37 in only the White participants, *F*(1, 18) = 10.50, *p* = 0.005. The sleep by race interaction also increased in effect size from η_p_^2^ = 0.17 in the overall sample to η_p_^2^ = 0.39 in the White participants, *F*(1, 18) = 11.64, *p* = 0.003.

## Discussion

To promote social justice, society must address violent behavior, police brutality, and racial discrimination. The current data suggest that some of these behaviors may be exacerbated by sleep loss, even when the sleep restriction is relatively mild (6.2 h/night), even when sleep restricted individuals believe they have “adapted” to the short sleep, and even in individuals with inclinations toward behaving in a socially desirable manner.

The current experimental findings provide causal support for an earlier correlational study in newly recruited police officers^21^. In Ma et al.’s^21^ study, newly recruited officers with self-reported total sleep time − 1 SD below the mean (≤ 5.25 h) showed greater racial bias in the shooter task than officers with self-reported total sleep time + 1 SD above the mean (≥ 8.05 h). Both their study and the current results implicated sleep loss in more “itchy trigger finger” type responding. A distinction in the studies, however, was that Ma et al.^21^ found that short sleepers were more likely to show a lenient decision criterion toward Black targets, whereas we found sleep restriction affected the decision criterion primarily for White targets. In another recent study^25^, total sleep deprivation (“all nighter”) in college student participants led to more errors on the Police Officer’s Dilemma Task, particularly more “shooting” of unarmed targets (but not in a racially biased manner). These effects were not mitigated by caffeine consumption. The differences across these data sets and our results could be due to study differences in experimental/correlational design, sample size, sleep measurement, type of sleep loss (acute, chronic), sample composition (e.g., young adults tend to be more open and liberal), or other factors that might become evident with further studies.

Our findings also join a growing literature that implicates short and poor-quality sleep in stereotyping and bias toward out-groups^13,16–18^, perhaps in a circadian-sensitive manner^19,20^. Sleep deprivation might lead to increased manifestations of discrimination by altering frontoparietal attentional processes^9,10^, amygdala/emotional reactivity processes^8,14^, and/or threat perception processes^15,26^. The first potential mechanism—altered frontoparietal attentional processes—relates to Ghumman and Barnes’^13^ idea that resources are needed to maintain control over prejudices, and if such resources are depleted by sleep loss, then biases and discriminatory behaviors will manifest. This idea is logical, but we did not observe direct evidence for it in the current study. When we controlled for performance on psychomotor vigilance and fluid intelligence tasks, we continued to observe that sleep restriction affected the decision criterion to shoot.

Distinguishing the amygdala/emotional reactivity and threat perception accounts is trickier because these views predict fairly similar outcomes. For example, sleep loss may make one react more emotionally to negative stimuli, manifesting in this paradigm as “shoot” responses on armed target trials. However, if threat perception is elevated after sleep loss, then “shoot” responses should increase across both armed and unarmed target trials. The collective data on decision criterion, therefore, favored the altered threat perception account. Nonetheless, we cannot dismiss the possibility that sleep loss also affects the decision criterion to shoot via amygdala/emotional reactivity, inhibitory inefficiency, impulsivity, altered ethical decision making, or general tendencies toward aggression^26–32^, accounts which are not necessarily mutually exclusive with the altered threat perception account.

The purpose of the current study was to investigate the causal effects of sleep loss on socio-emotional-cognitive processes that could contribute to racial discrimination. Another emerging literature that connects sleep and racial discrimination falls under the umbrella of health disparities. *Health disparities* refer to group differences in disease rates/outcomes that are systematic, avoidable, and unfair^33^. For example, the Centers for Disease Control and Prevention^34^ report that Black or African American individuals have considerably higher rates of diabetes, hypertension, heart disease, and HIV infection than White and Asian American individuals (disparities are also observed for Hispanic/Latinx individuals as well as American Indian/Alaska Native individuals)^35,36^.

One of the leading models for explaining health disparities points to *upstream determinants* influencing *downstream determinants. Upstream determinants* refer to fundamental social/environmental causes, including differences in socioeconomic status, discrimination, and other societal inequities. *Downstream determinants* refer to the individual’s experiences and can include allostatic load, self-determination, and health behaviors (note that models also indicate that gene-environment interactions feed back to upstream factors)^37,38^. Pertinent to the present work, racial/ethnic differences in sleep health are recognized as markers of health disparities (i.e., *sleep disparities*)^39–47^. Furthermore, sleep disparities are emerging as an important contributor to race/ethnic disparities in cardiovascular^48–52^, metabolic^52,53^, and cognitive^54,55^ health outcomes. Clearly, sleep health is important in all communities, even when isolated from matters of violence and aggressive race-based discrimination.

One of the limitations of the current study was the sample size, including a limited number of participants from non-White groups. On the one hand, this limitation does not undermine the primary purpose of understanding how majority groups (White participants) display racial biases and altered behaviors under mild sleep restriction (note that police officers in the U.S. are disproportionately White relative to Census estimates^56^). On the other hand, the limited variability in participant demographics hindered us from examining whether effects were driven by in-group/out-group determinants^57,58^ versus general stereotyping of Black men^59^. Increasing diversity in participant enrollment is a longstanding problem in both psychological and medical research^60–62^. Overcoming barriers to participant representation requires additional time and monetary resources for recruitment, collaboration with community leaders, and cultural tailoring^62,63^. It also requires increasing representation at the level of scientists, editors, and funding agencies^64,65^. In other words, academia as a whole must acknowledge and work to address its own systematic biases.

## Conclusions and future directions

Police work is one of the most sleep-depriving professions^5,6^. It is also one of the few professions that involves carrying firearms and encountering potentially hostile situations. It remains an open, empirical question whether the gun-safety training offered to police officers shields them from the deleterious effects of sleep loss. Though that is possible, we note the following empirical evidence: (1) shielding was not observed in a correlational study on sleep and shooter bias in newly recruited police officers^21^; (2) night shift patrol officers were less likely to prevent deadly outcomes in video-based use-of-force simulators than day shift patrol officers^66^; (3) police officers sleep even worse than the general public^5,6^; and (4) workers in other fields that require extensive, safety-focused training (e.g., medicine) still show susceptibility to sleep loss and shift-work fatigue^67–70^. Therefore, sleep loss deserves closer consideration by public officials and law enforcement as an insidious culprit underlying people’s sometimes faulty judgments and impulsive behaviors.

To eliminate discriminatory behaviors in society—including the shooting of unarmed Black individuals—there will be no substitute for eliminating underlying racism within individuals and systems. To achieve this end, education, policy changes, and individual efforts will be key. But, we also contend that there is room for reducing sleep debt in society^3,71,72^. Workplace sleep education programs^73^, behavioral change programs^74^, targeted memory reactivation interventions^75^, culturally tailored and personalized programs^76–78^, and schedules allowing for circadian alignment^79^ have all been shown to be effective. Based on the current results, as well as the literature on health disparities, it appears that correcting sleep loss in society may play a significant role in saving lives, including Black lives.

## Methods

### Participants

Forty-six adult participants were recruited through flyers and social media advertisements in spring 2018. The sample size was determined a priori to detect a large condition effect size, as often observed in sleep restriction studies^16,80^. They received monetary compensation for participating. Advertisements referred to the study as examining Sleep and Social Cognition to prevent expectation effects that would have emerged if the study were advertised as testing racial biases. Exclusion criteria included being under age 18 or over the age of 30, being a habitual short sleeper (< 7 h per night), and having clinically-diagnosed sleep disorders. Two participants did not complete all study sessions. Because there were only four African American participants we were unable to examine in-group versus out-group effects in this study; therefore, we excluded those data from the Police Officer’s Dilemma Task analyses (their data were included in other measures collected as part of this larger sleep study^22^). The study was approved by the Baylor University Institutional Review Board and all research was performed in accordance with relevant guidelines/regulations. All participants signed informed consents prior to the study and were debriefed upon study completion.

### Overview of design

The 5-day study consisted of two 1.5 h in-lab experimental sessions – one on Monday and one on Friday. For the four nights between each session, participants were instructed to maintain a wake time of 7:30 am and were randomly assigned to maintain a bedtime of either 10:30 pm (normal sleep condition) or 1:30 am (restricted sleep condition). To promote ecological validity, participants slept in their home environment (home-based and lab-based sleep restriction protocols produce similar effect sizes on cognitive functioning^80^). Across the week, we measured sleep using wristband actigraphy, which is designed to detect ambient light and movement (accelerometer-based).

### Equipment

The actigraphy device was the Actiwatch Spectrum Plus (Phillips Respironics Actiwach-2), which has been validated for distinguishing sleep/wake state against the gold standard of polysomnography^81,82^. The device was set to factory recommended settings that defined epoch length as 15 s, wake onset as 40 activity counts, and sleep as periods of rest with 10 min without movement. Participants also maintained a sleep diary^83^.

### Session 1

At Session 1 (Monday), participants completed a series of questionnaires. We measured recent sleep quality (Pittsburg Sleep Quality Index^84^), daytime sleepiness (Karolinska Sleepiness Scale^85^), social desirability^86^, and mood disturbances (Profile of Mood States^87^). Participants additionally completed three cognitive tests: the psychomotor vigilance test^12^, reading span^88^, and Raven’s Advanced Progressive Matrices^89^. At the end of Session 1, participants received the actigraphy device and sleep diary, and they were randomly assigned to the normal sleep and restricted sleep conditions. Assignment order was determined using the blocked random assignment method (blocks of 2, 4) using the sealedenvelope.com online tool. To minimize the risk of experimenter bias, the researchers who interacted with the participants were masked to each participant’s experimental condition. We accomplished masking by having a research staff member who would not be interacting with participants generate the random assignments and seal participants’ condition assignments in envelopes. Only after participants completed Session 1, did a research staff member hand the participant a sealed envelope with their condition with the instruction to open the envelope after leaving Session 1. Participants were explicitly instructed to not mention their assigned sleep condition to the experimenter as they were leaving or when they returned for Session 2. Participants were instructed not to nap during the week.

### Session 2

At Session 2 (Friday), participants returned to the laboratory at the same time as on Monday (typically, 8:30 am). We asked participants if they believed they had adapted to the sleep loss, and then re-administered the daytime sleepiness scale, mood disturbances scale, cognitive measures, and a medical error vignette task^22^. Lastly, participants completed the Police Officer’s Dilemma Task, administered via E-prime 2.0 software. In this task, participants are shown scenes with armed and unarmed men (targets) who are either White or Black^23,24^. The participant’s objective is to shoot (Q key) the armed targets and to not shoot (P key) the unarmed targets. Armed targets are holding either a revolver or a pistol, whereas unarmed targets are holding an aluminum can, a camera, a cell phone, or a wallet. In the procedure, empty scenes continuously flash in 500–1000 ms intervals, and after 1–4 empty scenes, a target scene will appear. After 16 practice trials, participants completed 100 experimental trials (25 White armed trials, 25 White unarmed trials, 25 Black armed trials, and 25 Black unarmed trials). To simulate the speeded nature of real world dilemmas, participants had to respond within 630 ms, otherwise the trial resulted in a timeout^90^. Response times were analyzed on correct trials. Accuracy data produced estimates for correct hits (shooting an armed target), misses (not shooting an armed target), false alarms (shooting an unarmed target), and correct rejections (not shooting an unarmed target). Following previous methods^24^, we subjected these accuracy data to signal detection analyses to produce estimates of *d*′, which reflects sensitivity, or the ability to discriminate armed from unarmed targets (which typically does not differ across White and Black targets^24^). We also generated estimates of decision criterion, or *c*, which reflects the threshold one sets for making a shoot response. Positive *c* values indicate a conservative threshold, such that one favors a don’t-shoot response; negative *c* values indicate a lenient threshold akin to having an “itchy trigger finger.” Signal detection estimates (*d*′ and *c*) were calculated separately for White targets and Black targets.

### Statistical analyses

Statistical analyses were completed using SPSS 26 (IBM) and graphically displayed using Prism 8.4 (Graphpad). D prime was calculated as *d*′ = zH = ZFA and criterion was calculated as *c* = − 0.5 X (zFA + zH). For both calculations, zFA refers to the z-score of false alarms and zH refers to the z-score for hits (with minimum and maximum set to 1/2n and 1–1/2n, following^24^). We conducted a series of 2 × 2 mixed analyses of variance (ANOVA) in which condition (normal, restricted) varied between subjects and target race (White, Black) varied within subjects. Alpha was set to 0.05 and all tests were two-tailed. Effect sizes were estimated using Cohen’s *d* and partial eta squared.

## Data Availability

Materials for the Police Officer’s Dilemma Task are publicly available (http://psych.colorado.edu/~jclab/FPST.html). Additional study materials and raw data are available at the Open Science Framework (https://osf.io/v9arg/).
